# Pilot Investigation: Older Adults With Atrial Fibrillation Demonstrate Greater Brain Leukoaraiosis in Infracortical and Deep Regions Relative to Non-Atrial Fibrillation Peers

**DOI:** 10.3389/fnagi.2020.00271

**Published:** 2020-08-28

**Authors:** Margaret E. Wiggins, Jacob Jones, Jared J. Tanner, Ilona Schmalfuss, Seyed Hossein Aalaei-Andabili, Kenneth M. Heilman, David J. Libon, Thomas Beaver, Catherine C. Price

**Affiliations:** ^1^Department of Clinical and Health Psychology, University of Florida, Gainesville, FL, United States; ^2^Department of Psychology, California State University San Bernardino, San Bernardino, CA, United States; ^3^Department of Radiology, University of Florida College of Medicine, Gainesville, FL, United States; ^4^North Florida/South Georgia Veterans Health System, Gainesville, FL, United States; ^5^Department of Internal Medicine, University of Florida College of Medicine, Gainesville, FL, United States; ^6^Department of Neurology, University of Florida College of Medicine, Gainesville, FL, United States; ^7^Departments of Geriatrics and Gerontology and Psychology, School of Osteopathic Medicine, Rowan University, Stratford, NJ, United States; ^8^Department of Thoracic and Cardiovascular Surgery, University of Florida College of Medicine, Gainesville, FL, United States

**Keywords:** atrial fibrillation, leukoaraiosis, white matter abnormalities, cardiac disease, aging

## Abstract

**Background:**

This pilot study explored differences in distribution of white matter hyperintensities (called leukoaraiosis; LA) in older adults (mean age = 67 years) with atrial fibrillation (AF) vs. non-AF peers measured by: (1) depth distribution; (2) anterior-posterior distribution; (3) associations between LA and cortical thickness; and (4) presence of lacunae and stroke.

**Methods:**

Participant data (AF *n* = 17; non-AF peers *n* = 17) were acquired with the same magnetic resonance imaging protocols. LA volume was quantified by cortical depth (periventricular, deep, infracortical) and in anterior and posterior regions. Cortical thickness by lobe was assessed relative to LA load.

**Results:**

Relative to non-AF peers, the AF group had twice the total LA volume (AF = 2.1% vs. Non-AF = 0.9%), over 10 times greater infracortical LA (AF = 0.72% vs. Non-AF = 0.07%), and three times greater deep LA (AF = 2.1% vs. Non-AF = 0.6%). Examinations of the extent of LA in anterior vs. posterior regions revealed a trend for more posterior relative to anterior LA. In the entire sample, total LA and infracortical LA were negatively associated with temporal lobe thickness. Only those with AF presented with lacunae or stroke.

**Conclusion:**

Aging adults with AF had more total white matter disease than those without AF, particularly near the cortical mantle and deep within the cortex. Total and infracortical white matter disease in the entire sample negatively associated with temporal lobe thickness. Results suggest that those with AF have a distinct pattern of LA relative to those without AF, and that LA severity for all individuals may associate with structural changes in the cortex.

## Introduction

Leukoaraiosis (LA), or white matter abnormalities seen on computed tomography (CT) or magnetic resonance imaging (MRI) scans (Hachinski et al., 1986), is a neuroimaging marker of vascular-related brain injury that can be associated with atrial fibrillation (AF). LA in the brain is at least two times greater in those with AF relative to those without AF (de Leeuw et al., 2000). This LA load discrepancy is believed to be the consequence of arrhythmia, increased thrombosis and embolization, and disrupted cerebral perfusion (Elhfnawy et al., 2019). To date, however, the *regional* specificity of LA for individuals with AF relative to non-AF peers and the relationship between LA and gray matter integrity remain largely unexamined with newer, sophisticated 3D volumetric quantification.

Research examining distinct regions of LA in participants with atrial fibrillation has had mixed results; however, some data suggest a potentially interesting pattern of abnormalities. When measured using a visual rating scale, those with AF have more LA near the cortex and in the deeper white matter relative to non-AF peers (Kobayashi et al., 2012). Another visual rating study suggests greater LA in anterior brain regions, such as around the frontal forceps, among patients with AF who also present with embolic stroke (Mayasi et al., 2018). Taken together, it may be that individuals with AF are particularly vulnerable to frontal lobe cortical thinning relative to non-AF peers who have little to no LA near the cortex (Price et al., 2012; Wiggins et al., 2019).

To explore whether individuals with AF have different white matter distributions from individuals without AF, the present study examined LA quantified as a percentage of white matter by cortical depth and in anterior and posterior regions for a sample of AF and non-AF peers with a sophisticated, 3-dimensional volumetric imaging and threshold measurement of LA. Given previous literature, we hypothesized that individuals with AF would have a greater LA volume than non-AF peers, particularly anteriorly and within the infracortical and deep regions of the brain. Additionally, the present study examined the association between LA and cortical thickness. Since LA appears to alter axonal connectivity, there may be a loss of neurons and intracortical integrity. Therefore, we hypothesized that greater LA load would be negatively associated with cortical thickness. Since LA may be caused by ischemia, we also explored group differences in the presence of strokes and lacunae volume.

## Methods

De-identified data and materials that support the findings of this study are available from the corresponding author upon reasonable request.

### Participants

Data were prospectively acquired as part of National Institutes of Health (NIH)-funded, University of Florida Institutional Review Board (IRB) approved investigations. Participants signed IRB-approved consent forms, and the study followed standards set forth in the Declaration of Helsinki and was in accordance with all institutional guidelines.

#### Recruitment

All participants had to meet the following inclusion criteria: English as primary language, intact activities of daily living (Lawton and Brody, 1969), and no signs of dementia based on neurobehavioral testing. Exclusion criteria for both groups included: history of head trauma with a loss of consciousness, neurodegenerative illness (e.g., Parkinson’s disease), documented learning or seizure disorder, less than a sixth-grade education, substance abuse in the last year, chronic medical illness known to induce encephalopathy such as major organ failure (e.g., liver disease), and implantable device precluding magnetic resonance imaging (MRI).

Individuals diagnosed with paroxysmal or persistent atrial fibrillation were eligible for the AF group. The AF group was enrolled as part of a clinical trial studying a minimally invasive thoracoscopic surgical procedure to treat atrial fibrillation. To be eligible for the surgical procedure additional exclusion criteria for the AF group included: age <18 or >80, presence of a left atrial appendage thrombus on CT or echocardiography, stroke fewer than 30 days prior to screening, ejection fraction <25%, previous thoracic surgery or empyema, left atrial diameter more than 55 mm, contraindication to anticoagulation with warfarin, mitral valve insufficiency (>2+) that would require open surgery, or prior cardiac surgery. The surgical subsample of the participants with atrial fibrillation are reported in a previous publication (Beaver et al., 2016).

Data from non-AF peer control group were acquired from a convenience sample of individuals who participated in a separate federally funded investigation with recruitment via local mailings, community fliers, and free community memory screenings. The non-AF peers completed the MR protocol with the same scanner and sequences. The control participants were selected to match the AF participants based on demographic characteristics (e.g., age, sex).

### Apparatus and Procedures

Each participant completed a general cognitive screening (Montreal Cognitive Assessment; MoCA; (Nasreddine et al., 2005) and a task of estimated premorbid intelligence (Wechsler Test of Adult Reading (WTAR; Wechsler, 2001) in combination with a basic neurobehavioral examination conducted by a neuropsychologist to rule out dementia. Health information was acquired through a background history questionnaire completed by a trained administrator. Using this information, a Charlson Comorbidity Index was calculated to quantify comorbidity level (Charlson et al., 1994).

#### MRI Protocol

For every participant, a Siemens 3T Verio scanner with an 8-channel head coil was used to acquire: (1) two T1-weighted scans (176 contiguous slices, 1 mm^3^ voxels, TR/TE = 2500/3.77 ms); (2) diffusion (two separate single-shot EPI, gradients applied along six directions (*b* = 100 s/mm^2^) and 64 directions (*b* = 1000 s/mm^2^), 73 contiguous axial slices, 2 mm^3^ voxels, TR/TE = 17300/81 ms); (3) T2-weighted 176 contiguous slices, 1 mm^3^ voxels, TR/TE = 3200/409 ms; and (4) Fluid Attenuated Inversion Recovery (FLAIR; 176 contiguous slices, 1 mm^3^ voxels, TR/TE = 6000/395 ms).

#### FreeSurfer Segmentation (Fischl et al., 2002; Fischl, 2012)

T1 cortical reconstruction and volumetric segmentation were performed with the FreeSurfer image analysis suite version 5.3 (documented and freely available for download), to acquire control variables of interest as well as cortical thickness values. An automated method from Freesurfer (Fischl et al., 2002; Fischl, 2012) provided variable MaskVol (Crowley et al., 2018) estimated total intracranial volume (TICV); TICV was defined as the sum of gray matter volume, white matter volume, and cerebrospinal fluid.

#### Leukoaraiosis (LA) Volume

From Fluid-Attenuated Inversion Recovery (FLAIR) scans, a reliable rater blinded to group and who had achieved intra-rater reliability and inter-rater reliability on LA measurement (dice similarity coefficient; DSC = 0.84 - 0.93; Inter-rater range = 0.80–0.83; DSC mean ± s.d = 0.84 ± 0.12) quantified LA with an in-house macro (Price et al., 2012) using ImageJ (Abramaoff et al., 2004) on FLAIR scans) LA voxels were thresholded, saved into 2D binary masks for each axial slice, and concatenated into 3D binary masks (Price et al., 2012). Dependent variable (DV) = LA mm^3^ relative to total white matter volume (LA Volume/White Matter Volume) (Price et al., 2012).

##### Depth of LA

Three areas of depth were *a priori* operationally defined (Price et al., 2015): (1) Periventricular LA: within 5 mm of the wall of the lateral ventricles. This boundary controlled for LA presence due to partial volume averaging (bending of the lateral ventricles which creates a halo within 2 mm of the ventricle wall or loss of ependymal cell layer due to reactive gliosis, which may implicate LA as a consequence of artifact or ventricular widening rather than vascular pathology); (2) Infracortical LA: within 5 mm of the internal cortical gray matter edge; hypothesized to disrupt U-fibers that travel in a tangential fashion connecting areas of the cortex to other proximal cortical regions; and (3) Deep LA: outside the infracortical and periventricular border zones and where the longitudinal fasciculi are located. To acquire depth of LA volumes as a percentage of regional white matter, masks were used to delineate regions of interest. For periventricular LA, ventricle masks were dilated by 5 mm. Original ventricle masks were subtracted from the dilated masks to create final periventricular masks. To create the infracortical region, FreeSurfer identified the gray matter-white matter boundary of the cortex and inflated the boundary by 2 mm relative to every point along the boundary region of white matter. Delineation of deep regions involved subtracting the infracortical and periventricular masks from the entire prosencephalon white matter mask. Next, the FLAIR images were skull-stripped using FSL BET (Jenkinson et al., 2012). The skull stripped FLAIR image was then co-registered with the FreeSurfer processed T1 image using FSL FLIRT (Jenkinson et al., 2002). The registration matrix was applied to LA masks, which were then segmented into three regional LA masks using *fslmaths*.

##### Anterior-Posterior LA

To examine LA within anterior and posterior anatomical boundaries, we subdivided the brain into anterior (frontal lobe only) and posterior (parietal and occipital lobes) regions. LA within each area was quantified as a portion of anterior/posterior white matter.

#### Ischemic Stroke and Lacunae Volume

A board certified neuroradiologist (IL) with extensive stroke rating experience (Price et al., 2012, 2014, 2015; Beaver et al., 2016) completed all stroke and lacunae measurements while blinded to group and any identifying medical or personal information. Ischemic strokes were measured on T1 and T2 sequences with volume calculated via the diameter approach in two-dimensions (Liem et al., 2007). The number of slices was multiplied by the slice thickness in the third dimension. Lacunar strokes were well-defined, dark lesions with a diameter ≥2 mm and <15 mm that held a stationary position between slices. Lacunae volumes were based on the formula of a sphere (4/3Πr^3^), with volumes from each brain summed to obtain an overall volume per participant. Lacunae were rated by region of subcortical gray matter structure (deep gray matter) and domain of white matter (periventricular, deep, infracortical) as seen on T1 and T2 sequences.

### Statistical Analyses

Data were checked for implausible values, missingness, and distributional form. Data were additionally assessed for normal or abnormal distribution using graphical displays (e.g., Q-Q plots, histograms). Due to non-normality, LA variables, stroke volume, and lacunae volume were log transformed (final skewness and kurtosis between −1, 1). We examined LA as a percentage of white matter (either total or region of interest). Independent samples *t*-tests examined group differences in age, education, cognition, stroke volume, lacunae volume, total intracranial volume (TICV), total white matter volume, and total LA. Chi-square analyses examined group differences in sex. Mixed-model ANOVAs covarying for TICV assessed within and between group differences in LA by depth and in anterior vs. posterior regions. Partial correlations correcting for TICV examined associations between LA variables of interest and cortical thickness values. All analyses were performed using SPSS version 24. The level of significance was set at 0.05. Effect sizes are interpreted as follows: *r* ≥ 0.1 = small, *r* ≥ 0.3 = medium, *r* ≥ 0.5 = large.

## Results

### Participants

Table 1. Groups were significantly different on the comorbidity health index (*p* < 0.001), with the atrial fibrillation group having more comorbidity in the domains of cerebrovascular disease, diabetes, and coronary artery disease. Groups were not significantly different in age, education, sex, general cognitive performance, estimated premorbid intelligence through WTAR, total white matter volume, and total intracranial volume (all *p* > 0.05).

**TABLE 1:** Demographics and volume of leukoaraiosis, white matter, total intracranial volume, stroke, and lacunae with mean (SD), minimum/maximum for atrial fibrillation (AF; *n* = 17) and non-atrial fibrillation peers (*n* = 17).

	AF	Non-AF	x^2^/t/u	*p*
Male: Female	11:6	11:6	0.00	>0.05
AF type*	9:8	–	–	–
AF duration	67.7 (92.6)	–	–	–
(months)	2/284	–	–	–
Age (years)	67.7 (8.0)	68.2 (4.8)	0.233	0.817
	47.0/78.0	62.0/79.0	–	–
Education	13.5 (2.9)	14.4 (2.0)	0.965	0.342
(years)	9.0/20.0	12.0/18.0	–	–
Charlson	1.8 (0.95)	0.24 (0.44)	6.26	0.000
comorbidity	1.0/4.0	0.0/1.0		
MoCA	24.3 (5.5)	26.8 (1.8)	181.5	0.194
	7.0/30.0	24.0/29.0	–	–
WTAR	37.6 (8.9)	38.6 (10.0)	0.328	0.745
	23.0/50.0	15.0/50.0	–	–
Leukoaraiosis	8507.7 (8445.2)	3985.8 (4707.1)	1.91	0.063
(mm^3^)	862.0/31616.0	241.0/19682.0	–	–
White matter	408015.7 (60602.5)	432537.5 (39577.2)	1.397	0.172
volume (mm^3^)	295197.0/515794.0	329177.0/502967.0	–	–
TICV (mm^3^)	1615269 (178016)	1605508 (137530)	0.179	0.859
	1322564.0/1923533.0	1358455.0/1842390.0	–	–
Stroke volume	28.4 (42.5)	0 (0)	2.759	0.009
(mL)	0.00/157.17	0.00/0.00	–	–
Lacunae	0.6 (1.9)	0 (0)	1.269	0.213
volume (mL)	0.00/8.00	0.00/0.00	–	–

*AF, atrial fibrillation; MoCA, Montreal Cognitive Assessment; WTAR, Wechsler Test of Adult Reading; TICV, total intracranial volume. ^∗^Persistent: Paroxymal.*

### Primary Aims

#### Leukoaraiosis: Total and by Depth

**FIGURE 1:**
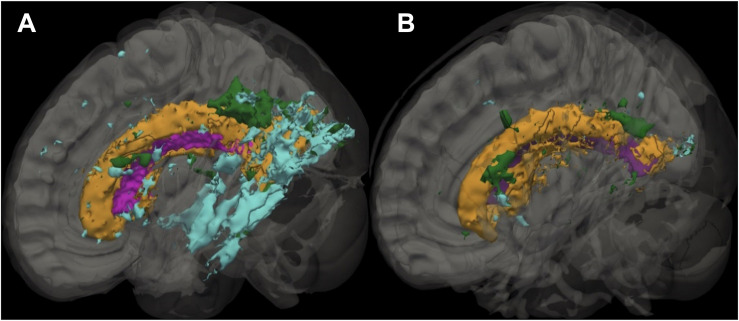
Sagittal slice showing the distribution of leukoaraiosis (LA) in periventricular, deep, and infracortical brain regions of individuals with atrial fibrillation (left; *n* = 17) and non-atrial fibrillation peers (right; *n* = 17). Representative participant brain for participants with AF **(A)** and non-AF peers **(B)** with the ventricle (purple) and leukoaraiosis segmented by depth: periventricular (orange), deep (green), and infracortical (blue).

**FIGURE 2:**
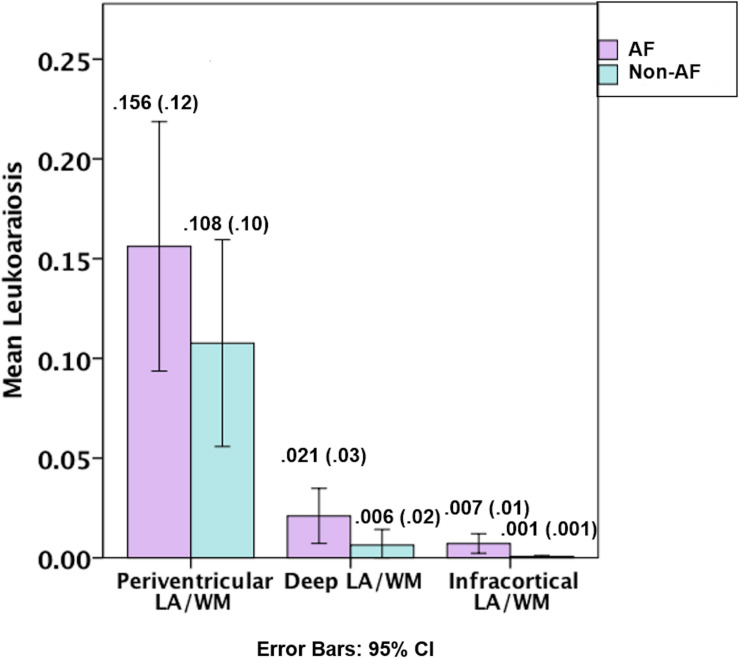
Graphical depiction of the distribution of leukoaraiosis as a percentage of white matter in periventricular, deep, and infracortical brain regions of individuals with atrial fibrillation (left; *n* = 17) and non-atrial fibrillation peers (right; *n* = 17). Mean (SD) for the percentage of leukoaraiosis within the white matter by depth for individuals with atrial fibrillation and non-atrial fibrillation peers.

Figures 1, 2, and Table 1. Those with AF had twice the amount of total LA than their non-AF peers (*p* < 0.05; 2.1 vs. 0.9%). A two group by three region mixed model ANOVA assessing depth differences in LA as a percentage of white matter after covarying for TICV showed a main effect for region [*F*(2, 62) = 8.243, *p* = 0.001; partial eta squared = 0.21] with more periventricular LA relative to deep or infracortical LA (*p*’s < 0.05). There was a significant region x group interaction [*F*(2, 62) = 7.22, *p* = 0.002; partial eta squared = 0.19]. Follow-up *t*-tests showed that AF patients had 10 times more infracortical LA [*t*(32) = −2.86; *p* = 0.007; 0.72 vs. 0.07%] and three times more deep LA [*t*(32) = −2.843, *p* = 0.008; 2.1 vs. 0.6%]; however, in the periventricular region there was no significant difference (*p* = 0.16; 15.6 vs. 10.8%).

#### LA in Anterior vs. Posterior Regions

Group by region (anterior/posterior) mixed model ANOVA showed a trend for differences by region [*F*(1, 31) = 3.10, *p* = 0.088; partial era squared = 0.09] with a trend for more posterior relative to anterior LA and no significant group x region interaction (*p* = 0.16).

#### LA Associations With Lobe Cortical Thickness

Analyses using partial correlations correcting for TICV demonstrated that temporal lobe thickness was significantly negatively associated with infracortical LA (*r* = −0.373, *p* = 0.035) and total LA (*r* = −0.372, *p* = 0.036) in the entire sample. LA was not associated with thickness in any other lobes (all *p* > 0.05).

### Stroke and Lacunae Volume

Table 1. Strokes were *only* present in the participants with AF (11/17; 65%; 28.44 ± 42.51), as were lacunae (9/17; 53%, 0.59 ± 1.92). Of the 11 participants with cortical strokes, four of these participants had subcortical strokes. Strokes were located in the deep white matter, deep gray matter, and in regions involving both gray and white matter in the frontal lobe and parietal lobe (e.g., complete chronic infarctions).

## Discussion

Our pilot exploratory analyses with volumetric scans and threshold imaging processes supports previous assertions that LA has a unique regional pattern in AF relative to non-AF peers. Yet, the pattern is not entirely confirmatory relative to previous reports (Kobayashi et al., 2012; Mayasi et al., 2018). Although statistically greater LA burden was indeed present in infracortical and deep cortical regions in patients with AF, both groups demonstrated a trend for more LA in the posterior region of the cortex which does not align with previous findings of greater anterior relative to posterior LA (Mayasi et al., 2018). Exploratory analyses in LA relative to cortical thickness also suggest that total and infracortical LA load were negatively associated with temporal lobe gray matter thickness (regardless of group), a region of the brain often impacted in neurodegenerative condition such as Alzheimer’s disease. This interesting finding suggests the need for further research regarding links between AF and neurodegenerative illness.

These data combined with previous reports (Kobayashi et al., 2012) suggest that AF presents with higher LA load near the cortical mantel. In this study, those with AF experienced 10 times more LA in the infracortical region than those without. U-fibers, also known as short or local association fibers, are the local connections between proximal brain gyri (Schmahmann and Pandya, 2007). With AF, decreased blood flow throughout the brain may contribute to dysregulated U-fiber integrity. Unfortunately, pathogenesis for infracortical LA is still speculative and particularly LA near the cortex. Animal models suggest LA is due to multiple pathways including hypoperfusion, inflammation, and blood-brain barrier changes (Hase et al., 2018). Molecular research also suggests that patients with history of stroke and AF who have periventricular and deep LA also have higher fibrinogen levels, a glycoprotein complex which plays a critical role in inflammation and coagulation (Wei et al., 2017; You et al., 2018). One study showed individuals with elevated fibrinogen levels (greater than 3.5 g/L) had 14-fold increase in periventricular and deep LA relative to individuals with normal fibrinogen measurements (Wei et al., 2017).

Contrary to previous reports that LA in those with AF is more anterior (Mayasi et al., 2018) our semi-automated imaging analyses suggest that while results did not reach significance, LA may be more commonly distributed *posteriorly* for both those with and without AF. Intracranial large artery atherosclerotic lesions in the posterior regions of the brain may have greater susceptibility to hemodynamic insufficiency, therefore leading to the pathogenesis of LA (Duan et al., 2018). In addition, small penetrating arteries supply more brainstem and thalamic brain tissue in the posterior relative to the anterior regions of the brain (von Sarnowski et al., 2017), which could lead to a greater vulnerability to the formation of posterior LA with disruption of lesions to the penetrating arteries.

We also found that individuals with more total LA and infracortical LA (regardless of group) showed greater bitemporal cortex thinness. We speculate this may also be due to the posterior watershed areas of the brain experiencing deep and infracortical disruption, or to decreased total brain perfusion due to hypertension leading to less blood flow to the temporal regions. Power analyses estimate a sample size of 42 participants per group will be needed for an effect of approximately 0.2. Future research needs to examine LA volume (total and infracortical) relative to temporal thickness in a larger sample size; findings may have relevance to neurodegenerative disease pathologies such as AD (Pasquini et al., 2016).

Regarding lacunae in AF, we replicate previous findings that lacunae were more commonly located in the deep white matter (internal capsule, superior to the trigone, parietal lobe), deep gray matter (putamen, globus pallidus, caudate, thalamus), and in regions involving both gray and white matter in the frontal lobe and parietal lobe (e.g., complete chronic infarctions). This is particularly important to consider in patients with AF, as LA and lacunae have both been shown to predict intracranial hemorrhage in patients with atrial fibrillation (Crosta et al., 2019). Greater severity and progression of LA are also associated with greater intracranial hemorrhage volume (Chen et al., 2018). Taken together, when working with older adults with cardiovascular conditions such as atrial fibrillation, it is important to consider the amount of LA and lacunae burden as potential prognostic information which can help inform treatment planning. Future studies with larger sample sizes should examine the associations between white matter disease distribution, cortical thickness, and lacunae and stroke volume. Although in our sample, general cognitive assessment via the MoCA and an estimation of premorbid intelligence via the WTAR were not statistically different between groups, we encourage future analysis of neuropsychological test performance, LA, and cortical thickness. Larger sample size studies examining cognition and LA may reveal pernicious neuroanatomical-behavioral patterns.

This pilot study is limited in the number of participants and availability of medical information regarding hypertension and medication dosage at time of testing that could contribute to LA presence. Strengths include a well-matched sample group, sophisticated neuroimaging protocols with isovoxel imaging and three-dimensional metrics of LA, and volumetric estimations as a percentage of white matter. The volumetric FLAIR sequences provided measurement of infracortical regions, an area potentially missed within previous studies using only visualization scales (You et al., 2018). Future studies are encouraged to apply volumetric imaging and threshold LA analyses to fully capture the regional distribution of LA and examine LA relative to thickness. Future studies should also aim to improve understanding of regional brain LA vulnerabilities to stroke risk profiles (Henninger et al., 2016; Dufouil et al., 2017), dementia risk (Chou et al., 2016), and neuropathological alterations including Wallerian degeneration of the U-fibers (Leys et al., 1991), amyloid-induced oligodendrocyte toxicity (Xu et al., 2001), and endothelial dysfunction (Szolnoki, 2007).

## Data Availability Statement

De-identified data and materials that support the findings of this study are available from the corresponding author upon reasonable request.

## Ethics Statement

The studies involving human participants were reviewed and approved by the University of Florida Institutional Review Board (IRB). The patients/participants provided their written informed consent to participate in this study.

## Author Contributions

MW: study conception, data analyses, and manuscript drafts. JJ, KH, and DL: study conception and manuscript drafts. JT: data collection, data analyses, and manuscript drafts. IS: data analyses and manuscript drafts. SH: data collection and manuscript drafts. TB: study conception, funding acquisition, data collection, and manuscript drafts. CP: study conception, funding acquisition, data collection, data analyses, and manuscript drafts. All authors contributed to the article and approved the submitted version.

## Conflict of Interest

The authors declare that the research was conducted in the absence of any commercial or financial relationships that could be construed as a potential conflict of interest.

## References

[B1] Abramaoff M. Magelhaes P. Ram S. (2004). Image processing with ImageJ. *Biophotonics Int.* 11 36–42.

[B2] Beaver T. M. Hedna V. S. Khanna A. Y. Miles W. M. Price C. C. Schmalfuss I. M. (2016). Thoracoscopic ablation with appendage ligation versus medical therapy for stroke prevention: a proof-of-concept randomized trial. *Innovations (Phila)* 11 99–105. 10.1177/155698451601100204 26914668 PMC6545892

[B3] Charlson M. Szatrowski T. P. Peterson J. Gold J. (1994). Validation of a combined comorbidity index. *J. Clin. Epidemiol.* 47 1245–1251. 10.1016/0895-4356(94)90129-5 7722560

[B4] Chen X. Jin Y. Chen J. Chen X. Cao X. Yu L. (2018). Relationship between white matter hyperintensities and hematoma volume in patients with intracerebral hematoma. *Aging Dis.* 9 999–1009. 30574413 10.14336/AD.2018.0108 PMC6284763

[B5] Chou R. H. Chiu C. C. Huang C. C. Chan W. L. Huang P. H. Chen Y. C. (2016). Prediction of vascular dementia and Alzheimer’s disease in patients with atrial fibrillation or atrial flutter using CHADS2 score. *J. Chin. Med. Assoc.* 79 470–476. 10.1016/j.jcma.2016.02.007 27234974

[B6] Crosta F. Desideri G. Marini C. (2019). Leukoaraiosis is an independent predictor of intracranial hemorrhage in patients with atrial fibrillation. *J. Thromb Thrombolysis* 47 527–532. 10.1007/s11239-019-01839-4 30877617

[B7] Crowley S. J. Tanner J. J. Ramon D. Schwab N. A. Hizel L. P. Price C. C. (2018). Reliability and utility of manual and automated estimates of total intracranial volume. *J. Int. Neuropsychol. Soc.* 24 206–211. 10.1017/s1355617717000868 28978362 PMC7111586

[B8] de Leeuw F. E. De Groot J. C. Oudkerk M. Kors J. A. Hofman A. Van Gijn J. (2000). Atrial fibrillation and the risk of cerebral white matter lesions. *Neurology* 54 1795–1801. 10.1212/wnl.54.9.1795 10802786

[B9] Duan W. Pu Y. Liu H. Jing J. Pan Y. Zou X. (2018). Association between leukoaraiosis and symptomatic intracranial large artery stenoses and occlusions: the Chinese Intracranial Atherosclerosis (CICAS) Study. *Aging Dis.* 9 1074–1083. 30574419 10.14336/AD.2018.0118 PMC6284759

[B10] Dufouil C. Beiser A. Mclure L. A. Wolf P. A. Tzourio C. Howard V. J. (2017). Revised framingham stroke risk profile to reflect temporal trends. *Circulation* 135 1145–1159. 10.1161/circulationaha.115.021275 28159800 PMC5504355

[B11] Elhfnawy A. M. Volkmann J. Schliesser M. Fluri F. (2019). Are cerebral white matter lesions related to the presence of bilateral internal carotid artery stenosis or to the length of stenosis among patients with ischemic cerebrovascular events? *Front. Neurol.* 10:919. 10.3389/fneur.2019.00919 31555196 PMC6727787

[B12] Fischl B. (2012). FreeSurfer. *NeuroImage* 62 774–781. 10.1016/j.neuroimage.2012.01.021 22248573 PMC3685476

[B13] Fischl B. Salat D. H. Busa E. Albert M. Dieterich M. Haselgrove C. (2002). Whole brain segmentation. *Neuron* 33 341–355. 11832223 10.1016/s0896-6273(02)00569-x

[B14] Hachinski V. C. Potter P. Merskey H. (1986). Leuko-Araiosis: an ancient term for a new problem. *Can. J. Neurol. Sci.* 13 533–534. 10.1017/s0317167100037264 3791068

[B15] Hase Y. Horsburgh K. Ihara M. Kalaria R. N. (2018). White matter degeneration in vascular and other ageing-related dementias. *J. Neurochem.* 144 617–633. 10.1111/jnc.14271 29210074

[B16] Henninger N. Goddeau R. P. Karmarkar A. Helenius J. Mcmanus D. D. (2016). Atrial fibrillation is associated with a worse 90-day outcome than other cardioembolic stroke subtypes. *Stroke* 47 1486–1492. 10.1161/strokeaha.116.012865 27217503 PMC4880452

[B17] Jenkinson M. Bannister P. Brady M. Smith S. (2002). Improved optimization for the robust and accurate linear registration and motion correction of brain images. *NeuroImage* 17 825–841. 10.1006/nimg.2002.1132 12377157

[B18] Jenkinson M. Beckmann C. F. Behrens T. E. J. Woolrich M. W. Smith S. M. (2012). FSL. *NeuroImage* 62 782–790. 21979382 10.1016/j.neuroimage.2011.09.015

[B19] Kobayashi A. Iguchi M. Shimizu S. Uchiyama S. (2012). Silent cerebral infarcts and cerebral white matter lesions in patients with nonvalvular atrial fibrillation. *J. Stroke Cerebrovasc Dis.* 21 310–317. 10.1016/j.jstrokecerebrovasdis.2010.09.004 21111632

[B20] Lawton M. P. Brody E. M. (1969). Assessment of older people: self-maintaining and instrumental activities of daily living. *Gerontologist* 9 179–186. 10.1093/geront/9.3_part_1.179 5349366

[B21] Leys D. Pruvo J. P. Parent M. Vermersch P. Soetaert G. Steinling M. (1991). Could wallerian degeneration contribute to “leuko-araiosis” in subjects free of any vascular disorder? *J. Neurol. Neurosurg. Psychiatry* 54 46–50. 10.1136/jnnp.54.1.46 2010759 PMC1014298

[B22] Liem M. K. Van Der Grond J. Haan J. Van Den Boom R. Ferrari M. D. Knaap Y. M. (2007). Lacunar infarcts are the main correlate with cognitive dysfunction in CADASIL. *Stroke* 38 923–928. 10.1161/01.str.0000257968.24015.bf 17272761

[B23] Mayasi Y. Helenius J. Mcmanus D. D. Goddeau R. P. Jr. Jun-O’connell A. H. Moonis M. (2018). Atrial fibrillation is associated with anterior predominant white matter lesions in patients presenting with embolic stroke. *J. Neurol. Neurosurg. Psychiatry* 89 6–13. 10.1136/jnnp-2016-315457 28554961 PMC5704976

[B24] Nasreddine Z. S. Phillips N. A. Bédirian V. Charbonneau S. Whitehead V. Collin I. (2005). The montreal cognitive assessment, moca: a brief screening tool for mild cognitive impairment. *J. Am. Geriatrics Soc.* 53 695–699. 10.1111/j.1532-5415.2005.53221.x 15817019

[B25] Pasquini L. Scherr M. Tahmasian M. Myers N. E. Ortner M. Kurz A. (2016). Increased intrinsic activity of medial-temporal lobe subregions is associated with decreased cortical thickness of medial-parietal areas in patients with Alzheimer’s disease dementia. *J. Alzheimers Dis.* 51 313–326. 10.3233/jad-150823 26836175

[B26] Price C. C. Mitchell S. M. Brumback B. Tanner J. J. Schmalfuss I. Lamar M. (2012). MRI-leukoaraiosis thresholds and the phenotypic expression of dementia. *Neurology* 79 734–740. 10.1212/wnl.0b013e3182661ef6 22843264 PMC4098873

[B27] Price C. C. Tanner J. J. Schmalfuss I. Garvan C. W. Gearen P. Dickey D. (2014). A pilot study evaluating presurgery neuroanatomical biomarkers for postoperative cognitive decline after total knee arthroplasty in older adults. *Anesthesiology* 120 601–613. 10.1097/aln.0000000000000080 24534857 PMC3930070

[B28] Price C. C. Tanner J. J. Schmalfuss I. M. Brumback B. Heilman K. M. Libon D. J. (2015). Dissociating statistically-determined alzheimer’s disease/vascular dementia neuropsychological syndromes using white and gray neuroradiological parameters. *J. Alzheimers Dis.* 48 833–847. 10.3233/jad-150407 26402109 PMC4724866

[B29] Schmahmann J. D. Pandya D. N. (2007). Cerebral white matter–historical evolution of facts and notions concerning the organization of the fiber pathways of the brain. *J. Hist. Neurosci.* 16 237–267. 10.1080/09647040500495896 17620190

[B30] Szolnoki Z. (2007). Chemical events behind leukoaraiosis: medicinal chemistry offers new insight into a specific microcirculation disturbance in the brain (a chemical approach to a frequent cerebral phenotype). *Curr. Med. Chem.* 14 1027–1036. 10.2174/092986707780362907 17439400

[B31] von Sarnowski B. Schminke U. Grittner U. Tanislav C. Bottcher T. Hennerici M. G. (2017). Posterior versus anterior circulation stroke in young adults: a comparative study of stroke aetiologies and risk factors in stroke among young fabry patients (sifap1). *Cerebrovasc Dis.* 43 152–160. 10.1159/000454840 28088807

[B32] Wechsler D. (2001). *Test of Adult Reading: WTAR.* San Antonio, TX: The Psychological Corporation.

[B33] Wei C. C. Zhang S. T. Liu J. F. Lin J. Yang T. T. Zhang S. H. (2017). Association between fibrinogen and leukoaraiosis in patients with ischemic stroke and atrial fibrillation. *J. Stroke Cerebrovasc Dis.* 26 2630–2637. 10.1016/j.jstrokecerebrovasdis.2017.06.027 28823490

[B34] Wiggins M. E. Tanner J. Schwab N. Crowley S. J. Schmalfuss I. Brumback B. (2019). Regional leukoaraiosis and cognition in non-demented older adults. *Brain Imag. Behav.* 13 1246–1254. 10.1007/s11682-018-9938-5 30128647 PMC6382599

[B35] Xu J. Chen S. Ahmed S. H. Chen H. Ku G. Goldberg M. P. (2001). Amyloid-beta peptides are cytotoxic to oligodendrocytes. *J. Neurosci.* 21:RC118. 10.1523/JNEUROSCI.21-01-j0001.2001 PMC6762453 11150354

[B36] You C. J. Liu D. Liu L. L. Liu Q. Q. Li G. Z. (2018). Correlation between fibrinogen and white matter hyperintensities among nondiabetic individuals with noncardiogenic ischemic stroke. *J. Stroke Cerebrovasc Dis.* 27 2360–2366. 10.1016/j.jstrokecerebrovasdis.2018.04.025 29773351

